# All We Need Is Trust: How the COVID-19 Outbreak Reconfigured Trust in Italian Public Institutions

**DOI:** 10.3389/fpsyg.2020.561747

**Published:** 2020-10-02

**Authors:** Rino Falcone, Elisa Colì, Silvia Felletti, Alessandro Sapienza, Cristiano Castelfranchi, Fabio Paglieri

**Affiliations:** ^1^Trust Theory and Technology Group, Institute of Cognitive Sciences and Technologies, National Research Council of Italy, Rome, Italy; ^2^Goal-Oriented Agents Lab, Institute of Cognitive Sciences and Technologies, National Research Council of Italy, Rome, Italy; ^3^Evaluation Research Group, Institute of Cognitive Sciences and Technologies, National Research Council of Italy, Rome, Italy

**Keywords:** COVID-19, trust, public authorities, risk management, social norms, socio-cognitive dynamics

## Abstract

The central focus of this research is the fast and crucial impact of the COVID-19 pandemic on a crucial psychological, relational, and political construct: trust. We investigate how the consequences of the pandemic, in terms of healthcare, state intervention and impositions, and daily life and habits, have affected trust in public institutions in Italy, at the time when the contagion was rapidly spreading in the country (early March 2020). In this survey, addressed to 4260 Italian citizens, we analyzed and measured such impact, focusing on various aspects of trust. This attention to multiple dimensions of trust constitutes the key conceptual advantage of this research, since trust is a complex and layered construct, with its own internal dynamics. In particular, the analysis focuses on how citizens attribute trust to Public Authorities, in relation to the management of the health crisis: with regard to the measures and guidelines adopted, the purposes pursued, the motivations that determine them, their capacity for involvement, and their effectiveness for the containment of the virus itself. A pandemic creates a bilateral need for trust, both in Public Authorities (they have to rely on citizens’ compliance and must try to promote and maintain their trust in order to be effective) and in citizens, since they need to feel that somebody can do something, can (has the power to) protect them, to act at the needed collective level. We are interested to explore how this need for trust affects the attributional process, regarding both attitudes and the corresponding decisions and actions. The most striking result of this survey is the very high level of institutional trust expressed by respondents: 75% of them trust Italian public authorities to be able to deal with the COVID-19 emergency. This is in sharp contrast with the relatively low levels of institutional trust characteristic of Italy, both historically and in recent surveys. Moreover, the survey allowed the discrimination of several potential predictors for trust, thus emphasizing factors that, during this crisis, are exhibiting an anomalous impact on trust.

## Introduction

The great societal challenge presented by the COVID-19 pandemic has prompted extraordinary efforts to meet such a challenge, from public authorities, civil society, and the scientific community. Extreme policies for containment, mitigation, and co-existence with the virus have been implemented by the governments of most afflicted countries, as well as by relevant international institutions (e.g., the WHO and the EU). At the same time, scientific research worldwide has focused on tackling the many facets of this dramatic phenomenon, including its impact on social relationships and psychological well-being, as well as the key socio-cognitive factors in promoting effectiveness of the proposed countermeasures. Several of these studies have highlighted the crucial and complex role of trust in dealing with the COVID-19 pandemic.

Llewellyn (2020) puts it very succinctly and effectively: “in times of crisis, trust is the most important thing to consider if you want to communicate health advice.” This blanket pronouncement is well-supported by previous evidence: in their systematic review on the importance of trust when preparing for and during a pandemic, Siegrist and Zingg (2014) found confirmation that “trust in health agencies positively influenced people’s willingness to adopt recommended behavior.” In addition, among the five recommendations for crisis communication highlighted by the authors’ survey, two directly concern trust management: “the focus should be not only on trust but also on confidence, and establishing trust in health authorities before a pandemic occurs is important.” This latter point is also stressed by Lewnard and Lo (2020), with reference to the current pandemic: “The effectiveness and societal impact of quarantine and social distancing will depend on the credibility of public health authorities, political leaders, and institutions. It is important that policy makers maintain the public’s trust through use of evidence-based interventions and fully transparent, fact-based communication.” It is worth noting that this emphasis on evidence and transparency, albeit crucial, describes only part of the relevant socio-cognitive dynamics that affect trust in public institutions: in particular, it collapses trust to confidence in information sources and their credibility, while a crucial problem is also trust in the institution’s power to intervene, as well as trust in collective compliance with the proposed measures. Finally, in specific circumstances, interesting inversions in cognitive cause–effect relationships can occur, as widely studied in cognitive sciences and social psychology (e.g., Festinger, 1957; Koller, 1988; Kunda, 1990; Epley and Gilovich, 2016).

In fact, the relevance of trust for dealing with health emergencies is also linked to the limits of direct enforcement of the required behavioral change: without the active cooperation of the population, any drastic intervention is doomed to fail, because the desired behaviors (e.g., frequently sanitizing one’s hands, wearing a facemask, and keeping a safe distance from others) cannot be effectively monitored on the required scale and with sufficient frequency. In a broad and comprehensive survey of social and behavioral results to support COVID-19 pandemic response, Van Bavel et al. (2020) highlight how most measures needed to contain an epidemic are, by their very nature, difficult to enforce directly: this, in turn, makes trust in public authorities all the more relevant. Based on scientific evidence gathered during previous outbreaks, Van Bavel et al. (2020) argue that “trust in institutions and governments (…) may play an important role.” For example, trust in the Liberian government was correlated with decisions to abide by mandated social distancing policies and utilizing clinics for care during the Ebola outbreak. Trust was also related to decisions to adopt preventive measures such as Ebola vaccinations in the DRC. Conversely, a lack of trust in public health officials may lead to negative effects on utilization of health services. Reliable information and public health messages are needed from national leaders and central health officials. However, local voices can amplify these messages and help build the trust that is needed to spur behavioral change (Van Bavel et al., 2020).

These expectations on the positive role of trust in promoting adherence and compliance with preventive regulations and guidance are finding ample confirmation also in recent studies on the ongoing crisis, both within and across various countries. In a nationally representative survey conducted in Denmark during the COVID-19 pandemic (*N* = 1782), Olsen and Hjorth (2020) measured the respondents’ willingness to apply social distancing in order to reduce contagion: they found that both lower levels of political trust and lower generalized social trust are negatively associated with willingness to distance and that younger male respondents with the lowest levels of education and least political trust report lower willingness to distance. In a nationally representative survey of Italian adults (*N* = 3452) conducted between the 18th and 20th of March 2020, Barari et al. (2020) observed high levels of understanding and self-reported compliance with containment measures, and noted that “even those who do not trust the government, or think the government has been untruthful about the crisis believe the messaging and claim to be acting in accordance.”

Trust acts as a precious commodity both for institutions and for scientists, both of which are crucial actors in the public response to the COVID-19 pandemic. In a large-scale background analysis of European Social Survey data on 25 European countries (*N* = 47,802) focused on the COVID-19 epidemic from January 22 to April 14, 2020, Oksanen et al. (2020) found that institutional trust acts as a protective factor: countries with low levels of institutional trust prior to the outbreak (including Italy) experienced significantly higher mortality rates during the crisis; moreover, their governments introduced restrictions against contagion later than countries with higher levels of institutional trust (calculated as the delta between the date when the restrictions came into effect and when the first confirmed COVID-19 death was reported in that nation), which in turn contributed to the severity of their death toll. These results on the relevance of trust as a protective factor are in line with previous studies on other epidemics, e.g., Ebola, showing how people with higher institutional trust are more likely to follow the advice and guidelines given by the health authorities (Blair et al., 2017; Vinck et al., 2019), as well as investigating the interplay between scientific and non-scientific sources in modulating people’s trust in healthcare information (Falade and Coultas, 2017). As for trust in science, its role has been highlighted in a recent study by Plohl and Musil (2020): using structural equation modeling (SEM) on a sample of 525 international, English-speaking respondents, the authors investigated whether and how risk perception and norm compliance for the COVID-19 pandemic may be affected by several constructs, i.e., religious orthodoxy, conspiracy ideation, intellectual curiosity, and trust in science, all measured with validated scales. Their results indicate that trust in science is by far the most important factor in producing appropriate risk assessment and high level of norm compliance. At the same time, trust in science, as opposed to the tendency to believe in alternative non-official sources, has been observed to be deeply affected by polarization and homophily (Bessi et al., 2016).

Looking at the specifics of the COVID-19 pandemic, so far the most insidious threat posed by the virus has been the combination of the rapidity of its spread with the high number of patients requiring treatment in intensive care, resulting in unprecedented strain on the healthcare system of affected countries. This in turn has prompted an increasing number of national governments to adopt extreme measures to limit the spread of the virus, often imposing very demanding limitations on citizens’ basic rights (e.g., social isolation, lockdown, and quarantine) and with dire socio-economic consequences (e.g., job insecurity, rising unemployment, loss of revenues, and inequalities). In such a unique scenario, the relevance of studying citizens’ trust in public institutions is manifold: on the one hand, the effectiveness of these measures and the collective ability to overcome their costs is conditional on the compliance of the population, which in turn is affected by trust in institutions; for this same reason, institutions actively seek to promote citizens’ trust, as a means to achieve their prevention goals; on the other hand, the very nature of the current crisis is likely to affect and shape how citizens conceptualize trust, and such socio-cognitive impact of the COVID-19 pandemic needs to be understood. Indeed, the current crisis acts as a magnifying glass in highlighting the essential role of trust in our societies (trust as “vinculum societatis,” the bond of society, to borrow John Locke’s famous expression), both for the psychological well-being of individuals and for the effective functioning of institutions.

The study presented in this paper contributes to this fast-growing body of knowledge on the interplay between trust in institutions and the COVID-19 pandemic, by discussing the results of a large scale survey (*N* = 4260) conducted on Italian citizens between March 9 and March 14, 2020. At that time, Italy had the most active outbreak of the virus worldwide, and its death count was growing at alarming rates; at the same time, extreme prevention measures were still relatively recent and rapidly changing in nature, sometimes from day to day (e.g., on March 11 new restrictions were introduced by the Government, closing public places such as restaurants, pubs, and most shops). Thus, our data offer insight into a time window in which the phenomenon was already in its acute phase in medical terms, yet still novel and unexpected for the population: this offers a privileged vantage point to observe how a pre-existing construct, trust in institutions, was affected by a sudden and profound change in the everyday functioning of the whole country, by a complete (albeit hopefully temporary) re-representation of one’s role in society and in personal relationships, as well as in the relationship between citizens and institutions.

The survey was theoretically inspired by the socio-cognitive model of trust developed by Castelfranchi and Falcone (2010): we chose this theoretical framework because it provides a rich and nuanced description of various *reasons for trust*, thus allowing us to probe not only the degree by which Italian citizens expressed trust toward the relevant public authorities engaged in the response to COVID-19 but also on what grounds such attitude was based. Our purpose, however, was not to look for direct validation of the theoretical model, but rather to collect as many detailed data as possible on the rapidly evolving Italian response to the COVID-19 emergency, from the standpoint of institutional trust: in this sense, this study was mostly intended as explorative. In particular, we wanted to compare our results with the well-documented low levels of trust in institutions exhibited by Italians before the onset of the crisis, which some have associated with tardiness in responding to the COVID-19 emergency across various European countries (Oksanen et al., 2020): we intended to see whether such widespread distrust toward public institutions would be confirmed or subverted during the initial stages of the COVID-19 outbreak in Italy and to offer some insights and suggestions regarding the original and peculiar nature of any discontinuity in institutional trust that may be associated with the current pandemic.

Moreover, we intended to take a closer look at the cognitive and social factors responsible for trust toward public institutions in the face of pandemic threats: the survey was designed both to discriminate several potential predictors for trust, so that subsequent analysis would allow us to individuate the most relevant ones, and to facilitate comparison with the underlying theoretical model, thus emphasizing factors that, during this crisis, are exhibiting an anomalous impact on trust—either because they determine trust more intensely than usual (overcharged factors) or because their impact is minimal or non-existent (anesthetized factors). Indeed, a key hypothesis that we wanted to test concerns the impact of COVID-19 on the very nature of the institutional trust construct: not only the overall trust in public institutions is affected by the pandemic and how these institutions respond to it, but also *the determinants of trust in institutions change and adapt to this crisis*, in comparison with other situations. Desperate times require desperate measures, and desperate measures induce a drastic reconfiguration of the cognitive underpinnings of trust in institutions. Our survey was designed to collect data on such paradigm shift in how institutional trust was conceptualized by Italian citizens during the early stages of the national response to the COVID-19 pandemic.

## Materials and Methods

### Sample

We used a snowball sampling method to determine the respondents: we collected a large sample (*N* = 4260, 57% women, mean age = 46 years, range = 18–85 years, *SD* = 13.42), relatively well-balanced in terms of geographical provenance (33% Northern Italy, 39% Central Italy, and 28% Southern Italy and main islands), with a significant portion of respondents (30%) residing in the regions most affected by COVID-19 at that time (Lombardy, Veneto, Emilia-Romagna, Marche, and Piedmont). The relatively uniform geographical distribution of the sample among the three macro-areas of Italy, as well as the significant proportion of respondents from highly affected regions, allows interesting comparisons based on participants’ residence. Moreover, the introduction of more drastic restrictions by the Italian Government at the end of March 11, 2020, invites considering also this temporal dimension in analyzing the data: in this respect, it is important that a fairly large set of participants (*N* = 829) completed the survey after those new restrictions had been introduced. Finally, it should be noted that the mean educational level of participants is very high: almost three quarters of respondents have a degree (38%) or post-graduate specialization (34%). The main characteristics of the sample are synthetized in Table 1.

**TABLE 1:** Sample characteristics.

	Regions most affected % (30%)	Regions less affected % (70%)	Total %
**Gender**			
Male	45	42	43
Female	55	58	57
Total	100	100	100
**Age (Mean = 46)**			
18 – 29	19	11	13
30–39	23	18	19
40–49	23	24	24
50–59	21	28	26
60–69	11	15	14
>70	3	4	4
Total	100	100	100
**Educational level**			
Middle school	3	2	2
High school	24	27	26
University degree	41	36	38
Post-graduate specialization	32	35	34
Total	100	100	100
**Geographical provenance**			
Northern Italy	96	7	33
Central Italy	4	53	39
Southern Italy/islands	0	40	28
Total	100	100	100

### Survey Structure

Data were collected with a 57-item questionnaire, using a five-point Likert scale for most items: an English translation of the whole questionnaire is available in the Supplementary Materials. The questionnaire was based on the socio-cognitive model of trust developed by Castelfranchi and Falcone (2010) and explored participants’ opinions on five main dimensions, in relation to the current COVID-19 crisis in Italy:

1. The *competence* of public authorities, both in implementing the appropriate safety measures and in issuing behavioral guidelines for their citizens;
2. The *intentions* of public authorities regarding the containment of the Coronavirus, by means of both security measures and behavioral guidelines;
3. The *purposes and effectiveness* of the safety measures implemented by PAs; the perceived impact of safety norms on the participant’s life, and his/her perception of other citizens’ compliance to the norms;
4. The participant’s *overall trust toward public authorities* and their motivations, the factors that determine the participant’s trust; the sources of information he/she most uses and their perceived trustworthiness;
5. The participant’s *expectations on the crisis’ long-term effects on trust*, i.e., citizens’ trust toward public authorities, scientists, and modern societies’ development model, as well as trust between peer citizens.

The questionnaire was administered online using the Google Forms platform. The questionnaire fully complied with ethical guidelines for human subject research and participation was conditional on the preliminary approval of an informed consent by each subject; the compilation took an average time of 10 min. Data analysis was performed using the SPSS (version 22) statistical software: the collected data were first analyzed through correlation analyses (given the asymmetric distribution of most variables, we considered Spearman correlation values); secondly, given the high number of items in the questionnaire, we conducted a principal component analysis (PCA) on each sub-section of the questionnaire prior to running regression analyses on the aggregated data.

## Results

### Descriptive Statistics

Full details on descriptive statistics for each item in the questionnaire are included in the Supplementary Materials, differentiating also based on geographical factors (Northern, Central, and Southern Italy; more affected vs. less affected regions) and temporal boundaries (before vs. after the March 11 announcement of new restrictions by the National Government). Here, we report only the most relevant findings, prior to more in-depth analysis, and only in terms of aggregate data, since no significant differences emerged at this level between different areas and different dates (albeit some interesting patterns were detected via regression analysis, see Section “Regression Analyses”).

#### Relevant Public Authority

When asked to indicate which public authority is the most adequate to take decisions concerning the COVID-19 emergency (item 14 in the questionnaire), 72.8% indicated the National Government, 13.3% indicated the Civil Protection, 4.2% indicated the Presidency of the Republic, 3.6% indicated the Regional Government, 0.9% indicated the municipal authority, and 5.2% indicated others. Hence, the overwhelming majority (90.3%) of respondents consider pandemics as a matter of national concern, which should be primarily addressed by national authorities. This should be taken into account while interpreting all other results, since most of the attitudes expressed by participants regarding features of public authorities (competence, intentionality, trust, etc.) should be understood with reference to national institutions, unless otherwise specified. Moreover, it is remarkable that the Presidency of the Republic, which is mostly a moral authority, is seen as having a greater role than Regional Governments, in spite of their leading role in the healthcare system, which in Italy is organized on a regional basis. Equally significant is the fact that only 0.1% of respondents (within the broader category “Others”) indicated any kind of international entity, including the European Union, as having a primary role in facing a pandemic outbreak. In short, at this stage of the COVID-19 emergency, Italian citizens strongly believed that this pandemic was not to be prominently addressed by either regional or international authorities, but was rather mostly a matter of national concern.

#### Institutional Trust

**FIGURE 1:**
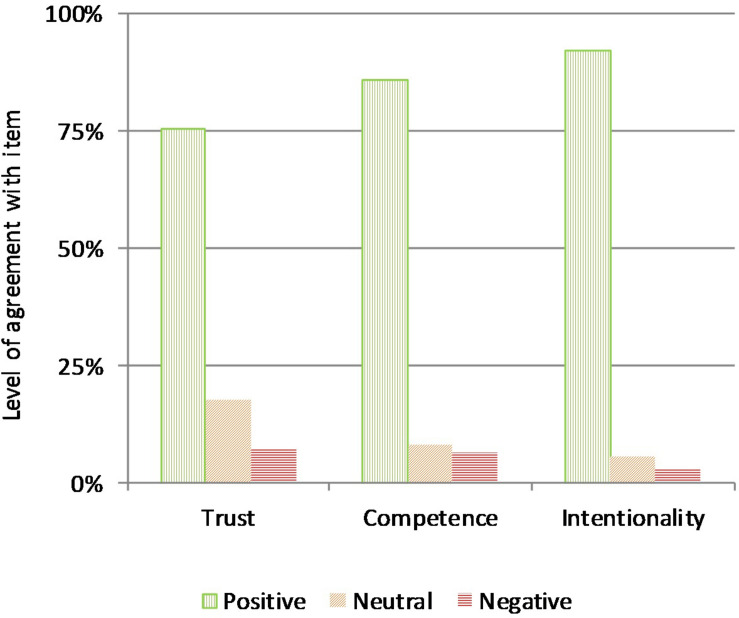
Trust, competence, and intentionality of the PA.

When asked to rank their overall trust in public authorities for the management of the COVID-19 emergency (item 33 in the questionnaire), 75% of respondents manifested either extreme (23.8%) or high (51.2%) levels of trust, 17.7% were non-committal, and only 7.3% expressed distrust (see Figure 1, left panel). As we will see in the section “Discussion and Conclusions,” these numbers are in sharp contrast, to say the least, with the average institutional trust reported for Italian citizens prior to the COVID-19 crisis, especially considering that the main target of this newfound trust was national public authorities (see above).

#### Competence

The competence of public authorities was assessed as their ability in planning both the right prescriptive measures (e.g., lockdown) and the appropriate behavioral guidelines (e.g., personal hygiene recommendations). On both counts, the majority of respondents expressed a positive belief in the public authorities’ competence (79.3% for measures, 82.7% for guidelines), whereas only a relatively small minority was either undecided (14.4% for measures, 11.4% for guidelines) or skeptical (6.3% for measures, 5.8% for guidelines). Moreover, correlational analysis indicates that competence scores for measures and guidelines are strongly and positively related (*R* = 0.738, *p* < 0.0001), suggesting that respondents did not really discriminate between prescriptive measures and behavioral guidelines, at least with respect to trust in public institutions: for this reason, in subsequent analyses, we collapsed these two items into a single competence value, calculated as the mean response for each subject to items 2 (competence on measures) and 3 (competence on guidelines) of the questionnaire (these are also the data reported in Figure 1, central panel). Other items in this section of the survey were designed to investigate the reasons behind participants’ beliefs on the public authorities’ competence: in summary, the overwhelming majority of the sample (91.8%) believed that it was the public authorities’ proper prerogative to take action and issue containment measures against the pandemic (item 4), and most respondents (71.7%) positively evaluated the use of experts’ advice by the public authorities during the COVID-19 crisis (item 5); there was instead less confidence in the organizational capacity demonstrated by public authorities in the early stages of the emergency (item 7: 44.8% expressed a positive evaluation, 33.6% were undecided, and 21.6% were critical), and the majority of the sample (54.3%) agreed that institutional communication on the COVID-19 presented some contradictions, either between different authorities or over time (item 6). In spite of these partial concerns, a significant majority of the sample (63.3%) did not express any skepticism on the competence of the public authorities in handling the emergency (item 8).

#### Intentionality

As for the competence, we inquired on the intentionality of public institutions separately for prescriptive measures and behavioral guidelines, asking participants whether they believed either type of intervention was both actively and honestly aimed at containing the COVID-19 pandemic. Again, respondents expressed an overwhelmingly positive belief in the good faith of public institutions, both in promulgating prescriptive measures (90.2%) and in issuing behavioral guidelines (89.1%): only a small minority was either undecided (7.1% for measures, 8.1% for guidelines) or skeptical (2.7% for measures, 2.8% for guidelines). Correlational analysis reveals again that intentionality scores for measures and guidelines are strongly and positively related (*R* = 0.794, *p* < 0.0001), further confirming that respondents did not really discriminate between prescriptive measures and behavioral guidelines, when it comes to assessing the public authorities’ trustworthiness in this emergency: hence, these two items on intentionality were collapsed into a unique intentionality value in subsequent analyses, using the mean response for each subject to items 9 (intentionality on measures) and 10 (intentionality on guidelines). Other items in this section of the survey were designed to investigate the reasons behind participants’ confidence, or lack thereof, in the nature of the public authorities’ intentions: in summary, we found confirmation of the fact that most respondents (72.1%) did not doubt that the intentions of the public authorities were consistent with their public statements (item 13), whereas a smaller majority (55.9%) considered the economic investment mobilized by the Italian public authorities sufficient to fight the pandemic (item 11: notice that only 16.4% considered it insufficient, with a significant portion of the sample, 27.7%, remaining undecided). Finally, asked whether other interests, e.g., political or economic, were at stake (item 12), the larger part of the sample (43.1%) answered in the negative, whereas 34.1% acknowledged the presence of such ulterior motives and 22.7% were unsure: as we will discuss further on, this question was probably easy to interpret in two markedly different senses—either negatively, as an accusation of having some hidden and problematic agenda, or positively, as the capacity to take into account all the key ramifications of the COVID-19 crisis, including its political and socio-economic aftermath. Overall, we registered strong confidence in the good faith of the intentions manifested by public institutions (Figure 1, right panel): this parallels the belief in the public authorities’ competence, and together, these attitudes support the high levels of institutional trust expressed by this sample.

#### Purposes and Effectiveness of the Public Authorities’ Intervention

Part of the survey was focused on the measures issued by public authorities as a response to the COVID-19 pandemic, in order to estimate both their perceived usefulness and the goal attributed to these interventions by the participants. The vast majority of our sample (85%) perceived these measures as being either useful (38.5%) or very useful (46.5%) in fighting the pandemic, whereas only a tiny minority was skeptical (2.6%), with the remaining 12.3% being undecided (item 15). When asked to assess the adequacy of the public authorities’ intervention (item 32), a more abstract notion involving a counterfactual comparison with alternative strategies, the majority rated current measures as adequate (53.8%), 33.2% were undecided, and only 13% considered them inadequate. In terms of the motivations associated with these measures, we asked participants to express agreement on three potential, non-mutually exclusive aims: reassuring the population (item 16), curbing the spread of COVID-19 (item 17), and creating unmotivated alarm (item 18). The vast majority (89%) agreed that the rationale of the public authorities’ intervention is indeed to contain the pandemic, whereas only 16.9% attributed to the public authorities the goal of reassuring citizens, and even fewer respondents (6%) regarded the proposed measures as a way of spreading unnecessary panic.

#### Impact of Containment and Beliefs on Compliance

**FIGURE 2:**
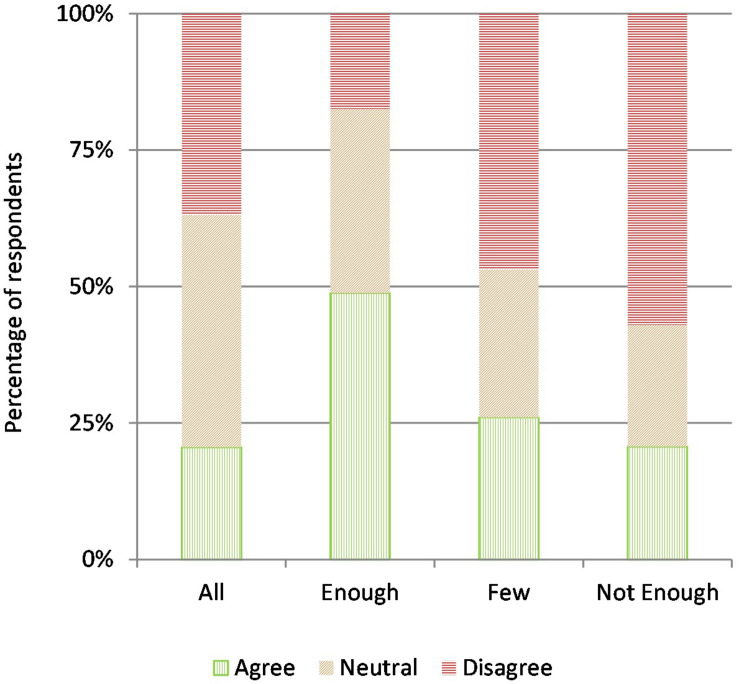
Expectations on compliance by others.

When rating the personal burden of the proposed restrictions on their own lives (item 19), 39% of participants expressed to feel a high level of impact, whereas 29.6% indicated little discomfort for the current situation and the remaining 31.4% reported medium levels of distress. However, regardless of the perceived impact on the public authorities’ intervention, the overwhelming majority of respondents agreed that such sacrifices were crucially beneficial for themselves and their families (item 20, 92.7% of agreement), for the society as a whole (item 21, 95.3%), and for both (item 22, 94.7%). Moreover, when asked to assess the usefulness of one’s personal contribution to these preventive measures, since they were intended for the whole population (item 23, a question aimed at implicitly measuring any “free-riding inclination” in our sample), as many as 96.6% of the participants considered their personal role relevant for the collective effort. Taken together, these data show that, albeit different people suffered more or less because of the containment measures, almost all agreed on their usefulness and on the necessity of personal sacrifice to deal with the pandemic: this suggests a mindset in which the shared goal of public safety trumps any individual concern, including personal discomfort, fear, and anxiety (an interpretation later confirmed by regression analysis, see section “Regression Analyses”). In terms of expectations on compliance with the sanitary restrictions by other fellow citizens (items 24–27), we observe a fairly varied pattern of response (see Figure 2): the most widespread belief (48.6% of agreement) is that enough Italian citizens, albeit not all, will comply with the regulations, thus making them effective (item 25); in contrast, there is skepticism both on the most optimistic scenario, i.e., full compliance (item 24, 36.9% of disagreement), and on the bleakest outcome, i.e., insufficient compliance (item 27, 56.9% of disagreement), although it is worth noting that pessimism is rejected much more strongly than optimism. The possibility that only few people will comply, and yet their efforts will be useful (item 26), is also rejected by the relative majority of the sample (46.9% of disagreement), yet interpreting this result requires caution, since it could either express skepticism on how many people will comply, or on the chances that limited compliance may indeed be useful. Regarding the motivations useful to induce compliance, we asked participants to express agreement on four possible motivational triggers: the expectation that everybody else will follow the new regulations (item 28), a personal concern for dangers (item 29), a spirit of collaboration in the face of the emergency (item 30), and trust in the fact that public authorities are doing everything in their power (item 31). All four motivations engendered significant levels of agreement, with the highest being the feeling of a common cause against a shared threat (90%), followed by trust in maximum effort by the public authorities (83.8%), concern for the associated risks (80.6%), and expecting others to comply as well (79.2%). It is interesting to note that a motivation tied to the collaborative dimension of trust in civil society, i.e., being united in pursuing a common goal, shows more than 10 percentage points of distance from a motivation inspired instead by the sanctioning view of trust, i.e., being able to monitor compliance by others, possibly to punish free-riders, as well as from fear of personal harm: this suggests that emphasizing collaborative motives (a strategy employed quite consistently by the Italian Government in its public communications during the early stage of the COVID-19 outbreak) may be more effective in promoting compliance than stressing individualistic goals.

#### Reasons for Trusting Public Authorities

This section of the survey asked respondents to provide a meta-cognitive evaluation of the most relevant factors promoting their trust in how public institutions are handling the COVID-19 crisis. Of the eight factors explored, the type of measures adopted by the authorities was the most frequently cited as important (item 41, 80.2%), followed by the information received on the crisis (item 36, 71.4%), the capacity of public authorities to actually enforce protective measures (item 35, 52.2%), the respondent’s profession (item 37, 46.9%) and his/her health condition (item 38, 40.6%), the opinions expressed by social relations such as friends and relatives (item 40, 37.3%) or colleagues (item 39, 35.9%), and the political connotation of the relevant authorities (item 34, 18.4%). Later on, we will use regression analysis to investigate the extent by which these self-reported data correspond to the relative weight of the actual factors affecting participants’ trust in institutions. For now, it appears that participants self-describe their theory of trust in fairly objective terms, giving priority to the factual nature of the proposed measures, the information they gathered (apparently with the exception of social channels; see below), and the extent by which public authority is able to enforce their recommendations; in contrast, relatively little weight is given to personal factors and social networks, and none at all to political partisanship. This last result suggests that the public response to the COVID-19 crisis was initially perceived as a matter of shared concern of all political parties, which in turn prompted a temporary truce in the usual partisanship characteristic of Italian politics; moreover, in their efforts to deal with the emergency, public authorities were regarded mostly for their institutional role, with little attention to their political affiliation (even when such authorities were the expression of certain political parties, as it was the case with the National Government). This interpretation also helps to explain the extremely high level of trust in public institutions with respect to the COVID-19 emergency during those few days, in a population well-known for its deep-seated distrust of politicians in general, and of political parties in particular: further analysis of this interesting anomaly will be presented in the section “Discussion and Conclusion.”

#### Information Sources

This section of the survey investigated both frequency of use (items 42–47) and perceived trustworthiness (items 48–52 and 54) of various types of information sources in relation to the COVID-19 pandemic, to get a better sense of what channels were most influential in affecting participants’ opinions on this topic; in addition, we collected data on the trustworthiness directly assigned to public institutions as information sources (item 53), which was high for 77.6% of the sample, average for 17.7%, and low only for 4.6% of respondents. With respect to other information channels, the data summarized in Table 2 highlight four main findings: (i) official online channels, e.g., the website of the Civil Protection, and scientists are both frequently used and considered reliable as information sources; (ii) in contrast, traditional media, albeit often consulted, are regarded as reliable only by less than half of our sample; (iii) family physicians are in general considered trustworthy, yet they are rarely used as information sources; (iv), finally, both social relationships and unofficial online sources, e.g., social media, are neither frequently used, nor widely believed. The result on unofficial online channels is especially surprising: whereas the very low credibility associated to these sources is understandable and even commendable, the fact that only one respondent out of four admits to using them frequently is hard to swallow, especially at a time in which personal contact was severely limited in Italy, thus making social media an even more attractive outlet for users. Besides, recent national statistics on Internet use in Italy do not agree with the picture painted by these data: according to the 2019 Global Digital Report^1^, compiled annually by WeAreSocial and Hootsuite, in 2019, 58% of Italian citizens were active social media users (with a growing trend with respect to 2018), and the average time spent on social media every day was a little less than 2 h per person. Besides social desirability effects (respondents may have been reluctant to admit gathering information via unofficial channels on such delicate topics), a possible explanation for this anomaly is in a common misperception of the role of social media as gatekeepers: someone who finds on Facebook a link to an article on a traditional newspaper, or is made aware by a post on Twitter of the latest press release on the official website of the Civil Protection, may be inclined to disregard the role of the social media in bringing these information to the user’s attention. Yet, this is how we use social media as information sources, often without even realizing it: we take advantage (or succumb, depending on the circumstances) of their agenda setting algorithms, which allow these platforms to act as powerful information brokers, rather than information producers.

**TABLE 2:** Use and reliability of information sources.

Source	Use			Reliability		

	Frequent	Average	Infrequent	Trustworthy	Neutral	Untrustworthy
*Traditional media*	78.7	11.9	9.2	41.7	38.7	19.6
*Official online channels (e.g., institutional websites)*	77.8	12.2	10	89.6	8.1	2.3
*Unofficial online channels (e.g., social media)*	25.6	18	56.5	4.3	17.7	78
*Family physicians*	24.6	20.1	55.2	63	26.3	10.7
*Scientists*	70.6	15.6	13.8	92.6	6.2	1.2
*Friends, relatives, acquaintances*	16.6	29.2	54.2	7.3	33.2	59.5

#### Expectations on Long-Term Impact on Trust

The final section of the survey intended to probe participants’ expectations on the long-term impact of the COVID-19 crisis on trust relationships between citizens and public institutions (item 55), between citizens and the dominant economic model of development (item 56), between citizens and the scientific community (item 57), and among citizens as peers (item 58). Here, the big winner is expected to be science: 72.8% of respondents believe that the current crisis will strengthen the trustworthiness of scientists as public figures. Expectations on the impact of trust toward public institutions and among citizens are less triumphant, yet still positive: 54.4% predict an increase in institutional trust after the COVID-19 pandemic, whereas 57% make the same prediction with respect to social trust, i.e., trust among peers. Finally, on future trust in the dominant model of economic development, our sample is evenly divided: 34% think that we will trust it more than before, 33.6% are undecided, and 32.4% expect an increase in distrust toward that model.

### Principal Component Analysis

As a preliminary step before running regression analyses, we used PCA to identify strongly correlated items in the data set and simplify the variables’ structure, in order to avoid multicollinearity issues in our regression models. Since the survey was theoretically motivated by the socio-cognitive model of trust (Castelfranchi and Falcone, 2010), we performed separate PCA on 10 subsets of items, to preserve relevant theory-based distinctions in the participants’ responses. Item 33, degree of trust toward public authorities in relation to the COVID-19 pandemic, was not included in the PCAs, since it was intended to act as the target of the regression models; we also excluded items 8 (doubts on public authorities’ competence) and 13 (doubts on public authorities’ intentions), since these were included in the survey merely as control questions for, respectively, items 2–3 and items 9–10; moreover, we kept separate from the PCAs item 19 (personal discomfort associated with public authorities’ measures), item 23 (usefulness of one’s own personal contribution to the collective effort), and item 32 (overall adequacy of public authorities’ measures), since we wanted to test their role as individual predictors in the regression models; finally, item 26 (expectation of very limited yet useful compliance by other citizens) was excluded for the PCA and regression analysis, due to the ambiguity in its interpretation already mentioned in Section “Descriptive Statistics.” The remaining 49 items led to the individuation of 21 principal components, as summarized in Table 3 (full details on the PCAs methods and results are provided in the Supplementary Materials). In order to be considered satisfactory, each PCA had to explain at least 50% of the cumulative variance, and further components were added only if they improved by more than 15% the explained variance.

**TABLE 3:** Principal component analysis results: from survey items to principal components.

*Section of the survey*	*Items considered*	*Principal components identified*
Competence of the PA	2, 3, 4, 5, 6, 7	Positive factors* (2, 3, 4, 5, 7) Negative factors* (6)
Intentionality of the PA	9, 10, 11, 12	Public safety intentions* (9, 10, 11) Other intentions* (12)
Aims of the PA intervention	15, 16, 17, 18	Contain* (15, 17) Reassure* (16) Alarm* (18)
Usefulness of personal sacrifices	20, 21, 22	Usefulness of sacrifices* (20, 21, 22)
Expectations on compliance	24, 25, 27	Universal compliance* (24) Sufficient compliance* (25) Insufficient compliance* (27)
Reasons for compliance	28, 29, 30, 31	Individualistic reasons* (29) Collectivist reasons* (28, 30, 31)
Reasons for trust in the PA	34, 35, 36, 37, 38, 39, 40, 41	Features of the PA (35, 36, 41) Personal and social variables (34, 37, 38, 39, 40)
Information sources: frequency	42, 43, 44, 45, 46, 47	Official sources (42, 43, 45, 46) Unofficial sources (44, 47)
Information sources: trustworthiness	48, 49, 50, 51, 52, 53, 54	Official sources and media* (48, 49, 51, 53, 54) Unofficial sources* (50, 52)
Future scenarios on trust	55, 56, 57, 58	Society (55, 57, 58) Development model (56)

The numbering used for items follows the order of presentation in the survey: the relevant items are from 2 to 58, since item 1 was the informed consent, whereas items 59–63 asked for demographic information. The asterisk (*) indicates principal components that were later used for regressions.

### Regression Analyses

In order to test our main hypotheses, we performed a multivariate regression model on raw data using IBM-SPSS 22 software. The dependent variable to be predicted was the overall trust manifested by participants toward public authorities involved in the COVID-19 response, i.e., item 33 in the survey. After some explorative iterations and based on theoretical considerations, we decided to include 22 independent variables in the final model: 15 principal components identified via PCAs (indicated with an asterisk in Table 3), 3 individual items that were conceptually independent from the other sections of the survey (personal discomfort associated with public authorities’ measures, usefulness of personal contribution to the collective effort, overall adequacy of public authorities’ measures), and 4 socio-demographic variables—age (coded as 1 = 18–40, 2 = 41–55, 3 = 56–85 years of age), educational level (coded as 1 = High school diploma or lower, 2 = University degree or higher), region of residence (coded as 1 = most affected regions, i.e., Lombardy, Emilia-Romagna, Veneto, Marche, and Piedmont, 2 = all other regions), and time of data collection (coded as 1 = before, 2 = after the March 11 new restrictions were announced). Preliminary analyses indicated that the respondent’s profession did not affect responses, so we excluded it from the model; as for gender, preliminary regressions showed no difference in the predictors of institutional trust between male and female respondents, so we excluded it from the final regression model and performed a separate set of analyses to assess its impact in our data (see Section “Gender Effects”).

We first run the regression analysis on the whole sample: the model had a good fit (*R* = 0.8) and explained 64% of the variance in the overall trust evaluation; 15 out of 22 independent variables were significantly correlated with trust (*p* < 0.05), and the most powerful predictors were positive indicators of competence of public authorities (β = 0.31, *p* < 0.001), perceived adequacy of the adopted measures (β = 0.174, *p* < 0.001), trustworthiness of official information sources (β = 0.145, *p* < 0.001), public authorities’ intention to contain the pandemic (β = 0.137, *p* < 0.001), and perception that public authorities’ efforts were focused on public safety, with no other agenda (β = 0.101, *p* < 0.001). All other significant predictors had an absolute value of β equal to or lower than 0.05. The non-significant predictors were personal discomfort due to the adopted measures, perceived usefulness of personal sacrifice, expectation of sufficient compliance (but notice that expectation of universal compliance was positively correlated with trust, whereas expectation of insufficient compliance was negatively correlated with it, both *p* < 0.005, suggesting an “all or nothing” attitude toward compliance), individualistic reasons for compliance (while collectivist reasons for compliance were strongly and positively associated with trust, *p* < 0.001), educational level, time of data collection, and age (the last one showed a marginally significant negative correlation, β = −0.018, *p* = 0.06).

We also applied the same regression model to subsets of participants, distinguishing first geographically (most afflicted regions vs. all other regions), then temporally (before and after the announcement of new restrictions by the Italian Government on March 11), in order to detect differences in how trust was processed depending on the severity of the sanitary emergency in various areas, and the strictness of the measures implemented by public authorities while the pandemic was still progressing. We already knew from descriptive statistics that no overall change in trust toward public authorities was observed across these contexts, yet we wanted to probe for more subtle differences, e.g., different predictors of trust, or different contribution of the same predictors, depending on region of residence and time of data submission. All β and *p*-values for the various multiple regressions are reported in Table 4; in what follows, we will focus only on the most relevant results.

**TABLE 4:** Multiple regression analysis: β and *p*-values for the whole sample, for region of residence (most affected vs. others), and for time of data submission (before vs. after new restrictions were announced on March 11).

	All		Most affected		Other regions		Before new restrictions		After new restrictions	
Predictors	β	*p*	β	*p*	β	*p*	β	*p*	β	*p*
Competence, positive factors	0.310	<0.001	0.352	0<.001	0.289	<0.001	0.311	<0.001	0.293	<0.001
Competence, negative factors	−0.048	<0.001	−0.042	0.01	−0.052	<0.001	−0.049	<0.001	−0.048	0.03
Public safety intentions	0.101	<0.001	0.113	<0.001	0.096	<0.001	0.103	<0.001	0.096	0.004
Other intentions	−0.050	<0.001	−0.038	0.03	−0.056	<0.001	−0.048	<0.001	−0.051	0.03
Intention to contain	0.137	<0.001	0.119	<0.001	0.146	<0.001	0.143	<0.001	0.108	0.001
Intention to reassure	0.023	0.02	0.012	0.47	0.029	0.02	0.023	0.03	0.016	0.47
Intention to alarm	−0.038	0.001	−0.049	0.01	−0.033	0.01	−0.040	0.001	−0.021	0.41
Personal discomfort	−0.006	0.54	−0.021	0.20	0.001	0.92	−0.005	0.60	−0.002	0.94
Usefulness of sacrifices	0.003	0.80	0.011	0.61	−0.003	0.86	−0.009	0.50	0.066	0.03
Impact of personal effort	0.030	0.01	0.020	0.34	0.036	0.01	0.023	0.09	0.062	0.03
Universal compliance	0.033	0.003	0.060	0.003	0.024	0.08	0.043	0.001	−0.006	0.81
Sufficient compliance	0.006	0.61	−0.047	0.02	0.026	0.05	0.008	0.54	−0.014	0.59
Insufficient compliance	−0.035	0.002	−0.051	0.01	−0.029	0.03	−0.024	0.04	−0.083	0.001
Individualistic reasons to comply	−0.005	0.62	−0.002	0.88	−0.007	0.55	−0.001	0.95	−0.024	0.30
Collectivistic reasons to comply	0.039	<0.001	0.069	<0.001	0.026	0.05	0.033	0.007	0.074	0.002
Adequacy of intervention	0.174	<0.001	0.146	<0.001	0.184	<0.001	0.171	<0.001	0.183	<0.001
Trustworthiness official sources	0.145	<0.001	0.134	<0.001	0.149	<0.001	0.153	<0.001	0.117	<0.001
Trustworthiness unofficial sources	−0.022	0.02	−0.019	0.25	−0.025	0.04	−0.034	0.002	0.029	0.19
Age	−0.018	0.06	−0.025	0.14	−0.015	0.20	−0.016	0.13	−0.027	0.22
Educational level	−0.007	0.44	0.000	0.98	−0.011	0.33	−0.004	0.73	−0.025	0.24
Time of data submission	−0.003	0.75	−0.017	0.28	0.003	0.78	N.A.	N.A.	N.A.	N.A.
Region of residence	−0.031	0.001	N.A.	N.A.	N.A.	N.A.	−0.035	0.001	−0.010	0.65

*Predictors are clustered based on their thematic similarity.*

Applying the model only to participants from the most affected regions in Italy at that time (Lombardy, Emilia-Romagna, Veneto, Marche, and Piedmont) revealed again a good fit (*R* = 0.825), explaining 68.1% of variance in trust assessment; the same model also had a good fit when applied only to participants from all other Italian regions (*R* = 0.788, 62.1% of explained variance). In both cases, the strongest predictors remained the same as in the whole sample, and also their order of importance was identical across regions, regardless of current outbreak severity (*p* < 0.001 for all the following predictors): positive indicators of competence (most affected: β = 0.352; other regions: β = 0.289), adequacy of the adopted measures (most affected: β = 0.146; other regions: β = 0.184), trustworthiness of official information sources (most affected: β = 0.134; other regions: β = 0.149), PA’s intention to contain the pandemic (most affected: β = 0.119; other regions: β = 0.146), and perception that public authorities’ efforts are focused on public safety, with no other agenda (most affected: β = 0.113; other regions: β = 0.96). In spite of the substantial similarity in how trust in public authorities was attributed by respondents in different areas of the country, some fine-grained distinctions emerge looking at those factors that were significant in one context but not in the other—and also exercising due caution, since a difference in significance does not necessarily imply a significant difference. In the most affected regions, we observed eight non-significant predictors, whereas there were only six in the other regions: four of these factors were irrelevant across both contexts (personal discomfort, perceived usefulness of the sacrifices, individualistic reasons for compliance, and time of data collection), whereas negative factors affecting competence of public authorities, intention to downplay the emergency, impact of personal effort, and trustworthiness of unofficial information sources were immaterial for respondents from the most affected areas, whereas they acted as significant predictors (albeit weak ones) for participants from other regions of Italy; in contrast, an expectation of sufficient compliance from other people had a significant negative correlation with trust in the most affected regions (β = −0.047, *p* = 0.02), whereas it had a marginally significant positive correlation with it elsewhere (β = 0.026, *p* = 0.05). Taken together, these results suggest that participants living in areas that were currently experiencing very severe outbreaks of COVID-19 had a more focused mindset when deciding whether to trust public authorities to deal with the emergency: less factors were considered relevant, and in particular, it was probably taken for granted that some inconsistency in public communication and intervention may occur, without necessarily jeopardizing trust (negative factors on competence), and that unofficial sources were not to be taken seriously when deciding whom to trust; at the same time, expecting that only a sufficient number of people would comply with the emergency measures had a negative impact on trust in public authorities, probably highlighting the fact that, in those regions, people believed that “enough is not enough”—that is, either everybody cooperates in facing the crisis (universal compliance) or we will not be successful in overcoming it. This extreme mindset is confirmed by the fact that the relevance of one’s own personal contribution did not affect trust attribution to public authorities in the most affected regions, whereas it did in other areas: this indicates again that collective compliance, not personal efforts, are perceived as the key to success by people currently facing the worst of the COVID-19 pandemic.

Looking instead for short-term shifts in trust assessment over time, in relation to relevant public events (i.e., the introduction of new measures by the Italian Government on March 11), we divided our sample based on time of data submission: before or after the public press release when the Prime Minister Giuseppe Conte announced the new restrictions to be implemented nationwide, to contain the COVID-19 outbreak. The model performed well across both time windows (before: *R* = 0.799, 63.8% explained variance; after: *R* = 0.806, 64.9% explained variance) and the strongest predictors remained the same, as well as their relative order of importance (*p* < 0.001 for all the following predictors): positive factors affecting competence of public authorities (before: β = 0.311; after: β = 0.293), perceived adequacy of the adopted measures (before: β = 0.171; after: β = 0.183), trustworthiness of official information sources (before: β = 0.149; after: β = 0.117), attributing to public authorities the intention to contain the pandemic (before: β = 0.143; after: β = 0.108), and the perception that their efforts were focused on public safety, with no other agenda (before: β = 0.103; after: β = 0.096). Again, we observed substantial stability over time in how trust in public authorities was attributed, with minor differences emerging only by comparing the significance and direction of some secondary variables. In general, the introduction of more severe restrictions had the effect of simplifying the metrics used to assess trust toward public authorities: before the March 11 announcement, only four variables failed to correlate significantly with trust, whereas after it, the number of irrelevant predictors increased to 8, indicating a more narrowly focused mindset in assessing the trustworthiness of the institutions in charge of dealing with the emergency. In particular, intention to downplay the emergency, personal discomfort associated with the proposed measures, and trustworthiness of unofficial information sources became irrelevant for trust in public authorities; unfortunately, the expectation of universal compliance also became equally irrelevant (before: β = 0.043, *p* = 0.001; after: β = −0.006, *p* = 0.81), while the negative correlation between expectation of insufficient compliance and trust was much stronger after the March 11 announcement (before: β = −0.024, *p* = 0.04; after: β = −0.083, *p* = 0.001). This suggests a turn for the worst in people’s expectations: before the new restrictions, trust was positively supported by expectation of universal compliance (the more I believe all others will behave responsibly, the more I trust the authorities), whereas after them, the influence of pessimistic fear became dominant (the more I doubt enough people will comply, the less I trust the authorities). As a possible reaction to this shift, it is worth noting that the positive correlation between impact of personal efforts in the COVID-19 response and trust in public authorities became significant only after March 11 (before: β = 0.023, *p* = 0.09; after: β = 0.062, *p* = 0.03), suggesting that the new measures strengthened in Italian citizens a sense of personal responsibility for the collective reaction to the virus. Finally, region of residence was a significant (albeit weak) predictor of trust before, but not after, the announcement of new restrictions by the Italian Government (before: β = −0.035, *p* < 0.001; after: β = −0.01, *p* = 0.65): this shows a stronger tendency to trust public authorities in the most affected regions before March 11, whereas this was no longer true after that date. Since overall trust in public authorities did not decrease after March 11 in the whole sample, this indicates a leveling in trust attribution across the country after the introduction of new measures, which in turn could be interpreted as a shift in the perception of the emergency: whereas in early March, a significant part of the Italian population still believed the outbreak to be somehow contained to specific regions, and thus a local problem unlikely to affect everybody in the same way, the nationwide interventions announced on March 11 made it crystal clear to all that COVID-19 was indeed a national concern.

Overall, these regression analyses show that, in Italy, trust in the capacity of public authorities to deal with the COVID-19 emergency was attributed in a fairly consistent manner during the time window of this survey (March 9–14, 2020) across different areas of the country, giving central prominence to positive indicators of competence in public institutions, assessing the adequacy of the proposed measures, verifying that proper intentions supported their application, and paying attention mostly to official information sources. All considered, this suggests a fairly reasonable and well-balanced judgment-making process for trust attribution, while the true anomaly remains the high levels of trust in public authorities recorded during the early stages of this emergency (see Section “Descriptive Statistics”), which are in sharp contrast with both long-term trends and recent surveys on institutional trust in Italy, prior to the COVID-19 pandemic. At a more fine-grained level, region of residence and time of data completion did reveal some interesting shifts in trust assessment, yet these insights should be interpreted carefully, since they concern relatively minor changes in the significance of secondary predictors, within a regression model with a high number of independent variables.

### Gender Effects

Comparing male and female respondents, a χ^2^ test revealed a small but significant difference (*p* = 0.004) in institutional trust in relation to the COVID-19 emergency: in particular, men were more likely to express high levels of trust toward public authorities involved in contrasting the outbreak (76.1% men vs. 74.3% women), whereas women were more often neutral (19.1% women vs. 15.7% men). Running the regression model described in Section “Regression Analyses” separately on male and female respondents showed that, although the main predictors remained the same (positive indicators of competence, adequacy of the measures, trustworthiness of official information sources, public intention to contain the pandemic, and institutional focus on public safety), age and region of residence were significant predictors only for women and not for men (AGE: women β = −0.026, *p* = 0.05, men β = −0.009, *p* = 0.516; REGION: women β = −0.052, *p* < 0.001, men β = 0.001, *p* = 0.964). To further investigate this interaction between gender and other socio-demographic factors influencing institutional trust during the COVID-19 emergency, we run a trivariate analysis on, respectively, gender × age × trust and gender × region × trust. The first analysis revealed that gender effects on institutional trust are significant (*p* = 0.038) only in the age range 56–85 years, which is also the most vulnerable to the virus: among respondents in this age range, the majority of those that expressed low levels of institutional trust were male (60%), whereas most of those neutral or highly trustful were female (59.4 and 53.8%, respectively). It is also worth noting that, after performing a bivariate analysis on the impact of age on trust, we found a highly significant effect (*p* < 0.001), with 86.1% of elderly respondents (56–85 years old) expressing high trust in public authorities, whereas this percentage drops to 69.6% for participants in between 18 and 40 years of age: this further confirms the role of vulnerability to the COVID-19 virus in eliciting higher attributions of trust, and it is consistent with previous findings on a negative correlation between age and willingness to comply with social distancing measures during the COVID-19 pandemic (Wirz et al., 2020). The second analysis showed that the relationship between gender and institutional trust is significant (*p* = 0.027) only in those regions that were most affected by the COVID-19 outbreak: in these areas, most of the respondents that manifested distrust in public authorities were men (55.5%), while the majority of the neutral and trustful participants were women (60.1 and 55.4%, respectively). Taken together, these results suggest that, whenever the situation was most critical (i.e., for the most vulnerable age range and in the most affected regions), men were overrepresented in the (small) group of people expressing distrust toward public authorities, whereas women were overrepresented among those neutral or trustful. Although this may suggest an interesting gender effect on resilience under extreme stress (women seem more likely than men to suspend judgment or look on the bright side, precisely when the situation is the most dire), it is worth noting that, regardless of gender, only a small minority of respondents were expressing distrust toward public authorities, even in the most affected age range (men 6.7%, female 3.8%) and in the most affected regions (men 10.8%, female 7.1%). Thus, these gender effects invite further investigation, but on their own, they do not justify any hasty conclusion on how different genders may react against health emergencies.

## Discussion and Conclusion

The most striking result of this survey is the very high level of institutional trust expressed by respondents: 75% of them trust Italian public authorities to be able to deal with the COVID-19 emergency. This is in sharp contrast with the relatively low levels of institutional trust characteristic of Italy, both historically and in recent surveys: according to the DEMOS & PI 22nd annual report on “The Italians and the State”^2^, based on a large representative sample (*N* = 1212) of Italian citizens over 15 years of age interviewed in December 2019, only 22% respondents trusted the State, whereas both Regional Governments (30%), European Union (34%), and municipal authorities (38%) fared better, while political parties were in the worst shape, with only 9 Italians out of 100 willing to trust them; in fact, of the main national institutions, the only one with decent levels of trust was the Presidency of the Republic (55%, still in sharp decline with comparison to 10 years before, in 2009, when it was as high as 70%). Also international estimates indicated relatively low levels of institutional trust: according to the Eurofound report on Eurofound (2018), Italians’ trust in the national government has been declining in the last few decades and is now below 20%, while the more recent data of the Eurispes Report–Italy 2020^3^, presented in February 2020, indicated trust in institutions at 14.6% (6.2 points lower than in 2019). Institutional trust in Italy in recent years is extremely weak not only in absolute terms but also in relation to other European countries: in their comparison of 25 EU states, based on data from the 2016 European Social Survey, Oksanen et al. (2020) reported very low levels of institutional trust in Italy, measured by respondents’ trust in five institutions (Parliament, politicians, political parties, the police, and the legal system); in fact, only Cyprus, Poland, Slovakia, and Bulgaria expressed stronger institutional distrust than Italy.

Moreover, this trend toward widespread distrust of public institutions is not a particularly recent feature of Italian politics: while in recent decades, it developed mostly against the backdrop of increasing tensions between populist movements and traditional political parties (Urbinati, 2019), massive erosion of public confidence in political figures was already ongoing in Italy well before the recent resurgence of populism worldwide—in the last decade of the 20th century, following the corruption scandals of Tangentopoli and its media resonance (Giglioli, 1996; Vannucci, 2009), and with the largely failed shift toward bipolarism during the Berlusconi age (Viroli, 2011). Even before that, a longitudinal analysis reveals that the confidence gap between electors and political institutions, characteristic of many post-WWII democracies, appeared in Italy much earlier than in other countries (Segatti, 2006)—so much so, that already in the 1960s LaPalombara (1965), a highly influential political scientist, described Italians’ attitudes toward politics with three emblematic words: alienation, fragmentation, and isolation.

Such a deeply rooted tradition of distrust in public institutions underscores the importance of the opposite trend registered in our survey, i.e., a sudden boost in institutional trust prompted by the COVID-19 crisis—a significant result that is also supported by other data collected in this survey, as seen in the “Results” section. Moreover, insofar as this newfound trust is grounded on trust in the expertise of the scientific authorities involved, it is also at odds with the widespread anti-scientific sentiment considered to be on the rise at the global level, variously stigmatized as “the death of expertise” (Nichols, 2017) and the crisis of epistemic deference (Marconi, 2019).

Surprising as it may be, there are several reasons to consider this finding on trust as reliable:

(i) Internal consistency: as discussed in Section “Results,” all other responses to the survey are consistent with a high attribution of trust to public authorities and indeed provide justification for such attribution.
(ii) External validation: just a few days after data collection for this study was concluded, a survey on a representative sample of Italian citizens (*N* = 1028, 16–17 March 2020) was conducted by the independent research center Demos & Pi^4^, providing substantial support to our main results: e.g., 71% trust both the Italian Government and the current Prime Minister, with 94% approval of the adopted measures, strong endorsement for the sanitary system (94%), the Civil Protection (88%), and the National Government (82%), coupled with lower levels of confidence in political parties (none of them above 30% of approval) and a rising skepticism toward the European Union (80% of respondents believe the Italian response to the COVID-19 emergency to be better than that of other EU countries, and only 35% consider the role played by the EU as positive in this crisis).
(iii) Low chances of social desirability effects: as demonstrated by the very low levels of institutional trust recorded in previous surveys, including recent ones, Italians have no qualms expressing public distrust toward public authorities—quite the opposite, in fact. Thus, there is no reason to assume that the current data on trust are inflated by social desirability effects.

Thus, there is a genuine phenomenon to be explained here: a veridical “trust boom” during the early stages of the COVID-19 crisis in Italy. The socio-cognitive theory of trust (Castelfranchi and Falcone, 2010) that inspired our survey provides the tools needed to craft a tentative interpretation of this remarkable fact, although the questionnaire itself was designed to record such a phenomenon, rather than explain it. Thus, the speculative nature of our interpretation cannot be stressed enough: our study revealed a highly significant and surprising phenomenon, for which now we look for an explanation. The interpretation we favor is the one that, to the best of our knowledge, appears more adequate to account for the pattern of results obtained in this survey; later on, we will contrast it with other alternative explanations and argue in favor of its superiority. Nonetheless, such interpretation remains tentative, and it is intended as a springboard and an inspiration for further studies that may either confirm or falsify it, rather than as something set in stone.

With this in mind, let us focus on the fact that trust, at its cognitive core, entails the decision to delegate to someone else (the trustee) the realization of a goal that is important to the agent who is expressing trust (the trustor). As a result, being able to choose not to trust someone requires either having alternative means to achieve the desired goal (e.g., “I will do it myself” or “I will delegate it to someone else”) or being ready to forsake that goal. However, neither of these options are available in the face of a pandemic: the relevant goal is personal and public safety, which is non-negotiable, i.e., it is not something we can decide to forget about, and the only course of action that offers reasonable chances of achieving it is to put our collective trust in public authorities, since there are no other available agencies we might appeal to (indeed, the only choice we have concerns the level of public authority we should confide in, and our sample clearly indicated the national level as the most pertinent one).

In other words, a pandemic like COVID-19 creates the preconditions for a collective case of *necessary trust in public authorities*, or institutional trust by force majeure: not in the sense that we are being manipulated by some hidden power, as some conspiracy theorists may be prone to believe, but because the very nature of the health crisis leaves us with no other option than to put our trust in public authorities (that is why we emphasize a need, a necessity for trust). It is worth noting that these pressures toward trust between citizens and public authorities in times of sanitary crisis are symmetrical: citizens have no alternatives to reliance in the relevant public institutions, yet these institutions themselves cannot help but trust in civic compliance to the proposed regulations, on pain of failure in containing the contagion, due to the limits of enforcement already emphasized in previous studies (Siegrist and Zingg, 2014; Lewnard and Lo, 2020; Olsen and Hjorth, 2020; Van Bavel et al., 2020). Necessary trust is a two-way street in health emergencies, for both citizens and public authorities.

Moreover, this two-way street is often cyclically traveled: in fact, the citizens themselves become fully aware (perceive the request and expectation) of the need for public authorities to receive the right degree of trust from citizens as a tool for achieving the *common* goal, and this awareness becomes one of the reasons for citizens to trust public authorities themselves. In other words, in the best-case scenario, this becomes a trust-based “alliance” toward a supreme common purpose. This civic alliance, or social pact, is grounded in a specific dynamic of trust: the trustor deliberately bestows trust on the trustee, even if partially skeptical of the trustee’s qualities, in an attempt of motivating the trustee to “rise to the occasion” and *become* trustworthy. This is the sense in which trust breeds trust, as noted both by trust theorists (e.g., Falcone and Castelfranchi, 2001b) and by political economists (e.g., Feld and Frey, 2002). In the context of the early stages of the COVID-19 pandemic in Italy, we suggest that Italian citizens put their trust in public authorities in charge of facing the crisis as a way of opening up a “trust credit line” and thus putting pressure on such authorities to prove themselves worthy of that credit. Similarly, public authorities frequently manifested full trust in citizens’ compliance with regulations (a topos often belabored on public occasions by all institutional actors, including the Prime Minister, the President of the Republic, and representatives of the Civil Protection), precisely for the same reason: by declaring their trust in the common sense and civic responsibility of Italian citizens, they were putting pressure on citizens to actually demonstrate such qualities.

Clearly, the objective need for trust created by a pandemic does not automatically evolve in greater trust toward public institutions. That need may find different outlets, so that other, bleaker outcomes may be equally possible: for instance, an already vulnerable trust relationship between citizens and public authorities may be shattered completely by a sudden crisis, especially if such crisis (or its poor management) are blamed on those authorities, possibly leading to a severe governmental crisis, and maybe even a takeover by authoritarian forces, or, in another scenario, public trust toward central authorities may dissolve, with citizens taking a turn toward tribalism and trying to face the crisis at the local level.^5^ While these options are certainly viable in general, our results suggest that neither of these paths was being seriously considered by most Italian citizens in early March 2020: our survey revealed a sudden increase of trust toward public institutions, rather than its collapse or further erosion, and that trust was directed toward national authorities, not toward specific charismatic leaders or local powers. According to our findings, faced with an unexpected need for public trust, the Italian people in early March 2020 opted for putting their trust (at long last) in their elected representatives at the national level, rather than turning to authoritarian figures or local authorities for solutions. Beyond the evidence of our data, how the management of the pandemic unfolded over those weeks provides further support to this interpretation. The Italian Government consistently acted as a mediator between all the social forces affected by the crisis, repeatedly demonstrating high reliance on the indications of the experts in crafting every containment measure: in short, the national authorities acted as the very antithesis of an authoritarian leader. At the same time, local authorities at all levels were relying on the guidance of the National Government for facing the pandemic and, in some cases, were actively asking for its direct intervention to solve a crisis that they were not equipped to deal with; more generally, there was widespread consensus, both in political debate and in the media, on the need for a national response to the COVID-19 emergency (a need well understood by our participants, as seen in the section “Results”). Again, an attitude that stands in sharp contrast with any shift toward tribalism.

Thus, assuming that the need for public trust prompted the high levels of institutional trust manifested by participants, we propose to interpret their other responses within the broad framework of motivated reasoning (Kunda, 1990) and cognitive dissonance theory (Festinger, 1957): as the chosen path to pursue the paramount goal of personal and public safety, trusting public authorities became in turn a necessary instrumental goal, thus coloring all other attitudes expressed by the respondents; more precisely, it prompted them to *actively look for reasons to justify their (unavoidable) trust in public authorities*, in order to minimize cognitive dissonance. Indeed, the need for trust experienced by Italian citizens during the COVID-19 emergency was at odds with their widespread attitude of distrust toward the very same public authorities they now needed to rely upon in the face of the outbreak: this, we argue, produced a massive and sudden shift in their perception of those public authorities, to better accommodate the new reality they had to deal with. In this interpretation, the trust boom observed in the survey was not produced by any collective epiphany on the actual qualities of the public institutions involved, but rather by a cognitive realignment of individual attributions to the current needs citizens were experiencing. All of a sudden, Italian citizens found themselves pressured to rely on some key public authorities in ways and to a degree never experienced before, at least since the worst days of World War II. Regardless of how well these authorities behaved in the first stages of the COVID-19 crisis, Italians opted to re-frame their attributional states in a way that made this novel institutional trust justified, thus flipping the usual causal connection involved in acts of trust: it is not a case of detecting the appropriate qualities in public authorities and therefore deciding to trust them, but rather an instance of having first the need to trust those authorities and then justify such trust by *assuming* that these authorities would manifest the qualities required to warrant that trust. This is also justified and supported by the implicit pact with which public authorities communicated the need for this responsible and trusted attitude toward them as decisive for the achievement of the common purpose.

It is worth noting that our reliance on motivated reasoning to explain some of these survey data is very different from the most common use of this notion in recent studies on public opinion: although originally conceived in much broader terms (Kunda, 1990), motivated reasoning in recent decades has become more and more associated with political ideology, with several studies investigating how partisan affiliations affect and filter our beliefs on matters of public interest (e.g., Redlawsk, 2002; Slothuus and de Vreese, 2010; Kahan, 2013; Bolsen et al., 2014). In fact, the same approach has been applied, with mixed results, to the public reaction to the COVID-19 pandemic, e.g., looking at how political partisanship affected people’s ability to discriminate between reliable information and fake news (Pennycook et al., 2020), timeliness in the adoption of restriction measures (Rosenfeld, 2020), health behaviors (Kushner Gadarian et al., 2020), and compliance with social distancing guidelines (Rothgerber et al., 2020) and stay-at-home regulations (Goldstein and Wiedemann, 2020). While the relevance of politically grounded motivated reasoning provides an interesting perspective on public opinion dynamics, other predictors have been found to be more relevant in explaining some of the target phenomena (e.g., fake news vulnerability, see Pennycook and Rand, 2019); more to the point, this is not the type of motivated reasoning we are discussing here. On the contrary, our data show no effect of political partisanship on trust attributions toward Italian public authorities in charge of coordinating the COVID-19 response, including those that did have a clear political connotation, e.g., the National Government. Instead, we appeal to the notion of motivated reasoning in relation to *a manifestly non-partisan goal*, i.e., public safety, and the related need to trust public authorities to be able to ensure such goal: this is the kind of motivated reasoning we argue influenced responses in our sample, independently from the political affiliation of either the survey participants or the relevant public authorities.

Alongside the preservation of consistency in citizens’ beliefs toward public authorities, there is also another, more emotional path through which a need for trust may generate broader shifts in public perception. As noted by many trust theorists (Luhmann, 1979; Gambetta, 1988; Batson, 1991; Hardin, 2002) and also described in the socio-cognitive model adopted here (Falcone and Castelfranchi, 2001a; Castelfranchi and Falcone, 2010), a fundamental function of trust is to allow both individuals and groups to face uncertainty, to moderate it and deal with it. Trusting someone or something immediately reduces the perception of risk; in this sense, trust offers the advantage of a subjective sense of safety, before and without being able to reach that safety objectively. It allows us to face the risk and take it, partially by giving us control over part of that risk, since trusting implies actively choosing to expose ourselves to a risk, i.e., the risk of having our trust betrayed (Mayer et al., 1995). This is why Koller (1988) individuated risk as a key determinant of trust, in the sense that a risky situation may bias people toward trustworthiness when assessing potential allies in facing such risk: “To the degree that the individual fears the occurrence of an event of negative valence (…) he exaggerates the subjective probability of an event of positive valence, which implies that he expects the interaction partner to behave promotively” (Koller, 1988, p. 275). This is very much in line with the higher levels of trust we observed in the most vulnerable age groups and in the Italian regions most affected by the COVID-19 outbreak (see Section “Gender Effects”). In the context of a health emergency such as the COVID-19 pandemic, this subjective dimension of trust becomes particularly apparent: consider how physicians and nurses in Italy turned overnight from marginalized workers in a distrusted field to the most revered national heroes. The individual and collective gain of this sudden change of perception is obvious: faced with the danger of contracting a deadly virus, the belief that your life will be in the hands of trusted professionals is incredibly valuable, not only for the unlucky few that will actually have to rely on those professionals, but for everybody, since it greatly helps in calming down their fear and anxiety. In this perspective, the trust boom recorded in our survey should be considered not only as a merely intellectualistic attitude but also as a response with deep emotional undertones: this is the type of trust that is not only cognitively justified, but also *felt*, insofar as it provides us with the calmness needed to remain productive under the extreme stress of a pandemic.

It is worth noting that emphasizing the motivated nature of institutional trust during a pandemic is not the same as treating this newfound trust in Italian public authorities as a fiction, just a desperate figment of the imagination of a population looking for solace from a terrible crisis. Nothing could be farther from the truth: precisely because this institutional trust was experienced as a matter of necessity by the Italian people, it is also genuinely (and dramatically) authentic. Italian citizens, during those terrible days in early March 2020, truly believed that public authorities would prove themselves worthy of their trust—possibly for the first time after many decades of increasing institutional distrust. Yet, it is a very fragile belief, because it is massively based on assumptions: should the public authorities subsequently fail to prove themselves equal to the task at hand, this huge “trust credit” would come due, producing an even bigger backlash in terms of the gap between citizens and institutions. This would indicate the clear failure of an “alliance” in which citizens have invested their trust in public authorities. On the other hand, an actual demonstration of trustworthiness by the public authorities during the COVID-19 emergency may engender a more durable and long overdue step change in institutional trust in Italy. As the Nobel prize Joseph Stiglitz put it in a recent interview to the Italian newspaper *La Repubblica*^6^ (30 April 2020), we should “not waste this crisis,” since it opens up genuinely new opportunities for rethinking the fabric of our societies. What is more, respondents in our sample were fairly optimistic on the future of trust relationships with their institutions, with scientists, and among themselves, while expressing reservations on the adequacy of the current economic model (see Section “Descriptive Statistics”). However, optimism is, by its very nature, a delicate thing, so the danger of experiencing a “trust crack” right after the initial trust boom is as real as ever.

Indeed, other ongoing research on the relationship between institutional trust and public response to the COVID-19 emergency may invite a bleaker outlook on how things will unfold: in their comparison of data from 25 European countries, Oksanen et al. (2020) highlighted a negative correlation between institutional trust prior to the crisis and the delay in introducing restrictions to curtail contagion—the less trust was manifested in public authorities before the COVID-19 outbreak, the more time passed after the first confirmed virus-related death and the introduction of containment measures. While we do not dispute the role of institutional trust as a protective factor against virus outbreaks (already well documented with Ebola, see Blair et al., 2017; Vinck et al., 2019), we are skeptical of the particular correlation observed by Oksanen et al. (2020), since it does not take into account the fact that different European countries were affected by the COVID-19 outbreak at different times: in particular, Italy, France, and Spain [all “late intervention countries,” according to Oksanen et al. (2020)] were among the first countries to record severe outbreaks, and much of the measures later adopted by other countries were largely based on the evidence coming in from these first, unwilling testbeds for the public response to the virus. This is confirmed by the same data used by Oksanen et al. (2020): in terms of absolute dates, Italy was among the first countries to endorse all the five types of interventions considered in their study, much earlier than many others that are instead regarded as “early adopters.” Moreover, the alleged correlation considers only the adoption of some form of interventions, without discriminating between countries that adopted all of them (like Italy) or just a few, sometimes even only one (as in the case of Sweden). This is probably why subsequent data do not seem to support the proposed correlation: for instance, Sweden, one of the countries with one of the highest levels of institutional trust before COVID-19, as of May 11, 2020 has a very high ratio to the number of deaths per million inhabitants (among the top six nations in the world); similarly, Belgium, where containment measures were adopted much more promptly than in Italy according to Oksanen et al. (2020), in early May 2020 had the world’s highest number of COVID-19 confirmed deaths per million inhabitants. For all these reasons, we are not persuaded that prior institutional trust was the main factor determining timely adoption of containment measures by public authorities: while early intervention remains critical in facing virus outbreak, in the case of COVID-19, we believe that this was determined mostly by other factors, e.g., where the outbreak manifested sooner in Europe.

Looking at the main predictors of trust highlighted by our regression analyses, respondents exhibited a matter-of-fact, evidence-based attributional strategy toward public authorities: consistently with the socio-cognitive model of Castelfranchi and Falcone (2010), competence, intentionality, trustworthiness as information sources, and the perceived adequacy of the proposed interventions were the most relevant factors in justifying trust in public authorities. The relevance given to the role of public authorities as information sources is also consistent with the significant weight that information has in shaping participants’ institutional trust, based both on their own self-report and on regression analysis (see Sections “Descriptive Statistics” and “Regression Analyses”): this highlights the importance of feedback and control for trust. Even when trust on public authorities is perceived as a necessity by citizens, they try to retain a measure of control over it, by monitoring the quality of institutional information channels. Equally suggestive are some of the factors that failed to impact institutional trust in our sample: most notably, the amount of personal sacrifice imposed upon participants by the restrictions introduced by the Government. Significantly, this dimension did not affect citizens’ trust in public authorities, contrary to what would be reasonable to expect under different circumstances: this, in turn, provides further support to our interpretation of the observed trust boom as a matter of necessity—insofar as public safety is the paramount goal, the severity of the necessary costs are immaterial in modulating institutional trust. This provides a nice illustration of the complex and context-dependent nature of feedback mechanisms on trust attributions: whether or not a certain observable feature of the situation (in this case, personal costs) will affect trust depends on its role within a broader attributional process, which cannot be oversimplified as a single feedback loop (for discussion, see Falcone and Castelfranchi, 2004).

Finally, it is worth stressing that the main predictors of trust remained stable both geographically and temporally: nonetheless, controlling for region of residence allowed us to notice a more focused mindset for trust attribution in the most affected regions, whereas comparing responses before and after the new restrictions introduced in Italy on March 11 highlighted a leveling effect of these measures, which made us realize the national character of the COVID-19 crisis to everybody, including citizens living in areas with only minor outbreaks.

This last point underscores a common pattern to many of our main results: *a shift from the particular to the general* in how institutional trust is granted and justified by citizens, apparently caused by the unique circumstances of the COVID-19 pandemic. As we discussed in section “Results,” the responsibility of dealing with this emergency was clearly assigned to the National Government, whereas regional and local authorities were perceived as marginal; moreover, high confidence was granted to public institutions, largely ignoring their political affiliation, unlike what happened in other countries, e.g., the United States (Goldstein and Wiedemann, 2020; Kushner Gadarian et al., 2020), and without concern for any further agenda they might serve (in fact, trust in public authorities was paralleled by distrust in the various political parties, including those currently in power); consistently with this mindset, collectivistic reasons for institutional trust trumped individualistic concerns, and the perception of a common effort toward shared goals overshadowed any personal sacrifice that may be required to individuals and groups (this also relates to the fact that personal health itself obliges to look and reflect primarily on collective health, on which the former strictly depends); finally, confidence in each other’s compliance with general rules was high, and the future outlook on trust was positive for public institutions, science, and civic society, not so much for the overall model of development. In short, participants responded to this survey not as individuals calculating trust based on likelihood of personal gains or losses (the standard economic view of trust), but rather as members of a collective subject, jointly engaged in facing a problematic situation.

This tendency to make common cause against a shared concern is one of the most valuable assets any society can leverage to fight a public crisis, so in this sense, our data paint a positive picture of how Italian citizens responded to the COVID-19 emergency, as far as trust in public authorities is concerned. However, as repeatedly stressed above, this asset is also incredibly delicate, especially in a country with a complex and thorny history of institutional distrust, like Italy. Hence, a crucial research priority for future research, both in the short run and in the long term, is to keep monitoring how trust dynamics between citizens and public authorities will be affected by the next stages of the COVID-19 pandemic: in fact, while our data suggest a generally positive reaction in the early phases of the emergency, they provide no guarantee of the fact that such trend will continue in the same direction. On the contrary, as mentioned, things could either turn for the best, as our respondents chose to believe, or turn for the worst, should public authorities fail to live up to their citizens’ high expectations.

## Data Availability Statement

All datasets generated for this study are included in the article/Supplementary Material.

## Ethics Statement

This study complied with all the ethical guidelines and standards for online surveys with human participants, in accordance with the local legislation and institutional requirements. The participants provided their written informed consent to participate in this study and were free to quit the survey at any time.

## Author Contributions

RF led the design of the survey. EC, AS, and SF performed data analysis. RF and FP wrote most of the manuscript. All authors listed have made a substantial, direct and intellectual contribution to the work, and approved it for publication.

## Conflict of Interest

The authors declare that the research was conducted in the absence of any commercial or financial relationships that could be construed as a potential conflict of interest.

## Footnotes

1 Source: https://wearesocial.com/it/digital-2019-italia

2 Source: http://www.demos.it/2019/pdf/5247itasta2019_20191223.pdf

3 Source: https://eurispes.eu/news/eurispes-risultati-del-rapporto-italia-2020/

4 http://www.demos.it/a01705.php

5 We are grateful to one of the reviewers for pointing out the importance of considering also these alternative scenarios.

6 Carofiglio, G. (2020, April 30). *Joseph Stiglitz: “Non sprecate questa crisi.”* La Repubblica, Retrieved from https://rep.repubblica.it/pwa/robinson/2020/04/30/news/joseph_stiglitz_non_sprecate_questa_crisi_-255286208/
